# Editorial: Milk and dairy products: Linking the chemistry, structure, processing, and food properties

**DOI:** 10.3389/fnut.2022.1103710

**Published:** 2022-12-06

**Authors:** Mutamed Ayyash, Gang Chen, Afaf Kamal-Eldin

**Affiliations:** ^1^Department of Food Science, College of Agriculture and Veterinary Medicine, United Arab Emirates University, Al Ain, United Arab Emirates; ^2^Institute of Quality Standards and Testing Technology for Agro-Products (IQSTAP), Chinese Academy of Agricultural Sciences (CAAS), Beijing, China

**Keywords:** milk, dairy products, chemistry, processing, properties

Milk and dairy products played a vital role as an important component of the diet of humans throughout history. Understanding the different milk constituents and their contribution to the structure, texture, sensory, and nutritional value of milk and dairy products is continuously increasing. At the same time, none-bovine milk sources are emerging and are foreseen to bring new qualities to existing products. The contribution of the new products to the consumer acceptability and nutritional value of milk products is an interesting topic to pursue. This Research Topic is particularly interested in cross-cutting research and new knowledge on the relation between milk composition, properties, and functionality.

This topic included six articles covering different aspects related to milk chemistry and related properties such as flavor, protein stability, and health benefits. Two studies are dedicated to the effect of feed on the aroma of raw bovine milk. The first study is a comprehensive study that identified 99 volatile aroma compounds in the milks of cows fed different diets using gas chromatography-mass spectrometry and gas chromatography-olfactometry. The study provided evidence that the diet significantly influences the abundance of the aroma of the milk and found that at least 33 compounds were transferred directly from the diet to the milk (Clarke et al.). In the second study, the effect of flaxseed supplementation on the volatile organic compounds in cow milk was studied using headspace-gas chromatography-ion mobility spectrometry (HS-GC-IMS). The results showed that although flaxseed supplementation significantly increased the n-3 PUFA in the milk, it had a very small effect on the volatile organic compounds contents (Huang et al.).

Paper 3 studied the effect of different treatments on the preservation of milk whey proteins. It found that the immunoglobulins (sIgA/IgA, IgG, IgM) in donor milk were preserved during freeze-thawing with homogenization, vat-pasteurization, while retort sterilization and ultra-high-temperature processing degraded them. Lactoferrin was reduced by freeze-thawing with homogenization, Vat-pasteurization, and UHT treatments by 35, 65, and 84%, respectively, while lysozyme survived all processing conditions (Liang et al.).

Most of the existing knowledge is based on bovine milk and products, but research on milks from other animal species adds new dimensions of knowledge. The focus is currently more on the health implications of these milks. The fourth paper evaluated the effect of supplementation of mice with the human milk oligosaccharide 2′-fucosyllactose on inhibiting intestinal inflammation by regulating gut microbiota, protecting goblet cells, and stimulating mucin secretion through TLR4-related pathway (Yao et al.). The fifth paper showed that koumiss made from horse milk modulated intestinal immune function in rats fed cyclophosphamide by increasing the number of leukocytes, repairing the tissue structure of the spleen and thymus, and increasing the CD4^+^/CD8^+^ ratio (Li et al.). The last paper reviewed and discussed the antidiabetic effect of camel milk and its potential mechanism of stimulating the insulin receptor and its related signaling pathways in HepG2 and HEK293 cells by lactoferrin and other whey peptides (Anwar et al.).

## Author contributions

All authors listed have made a substantial, direct, and intellectual contribution to the work and approved it for publication.

